# Microenvironmental Traits of Classical Hodgkin’s Lymphoma in Adolescents and Their Prognostic Impact

**DOI:** 10.3390/cancers16244210

**Published:** 2024-12-18

**Authors:** Clara Bertuzzi, Simona Righi, Giovanna Motta, Maura Rossi, Matteo Carella, Giulia Gabrielli, Elena Facchini, Maurizio Baldassarre, Arcangelo Prete, Pier Luigi Zinzani, Massimo Mascolo, Claudio Agostinelli, Elena Sabattini

**Affiliations:** 1 Haematopathology Unit, IRCCS Azienda Ospedaliero-Universitaria di Bologna, 40138 Bologna, Italy; clara.bertuzzi@aosp.bo.it (C.B.); giovanna.motta@aosp.bo.it (G.M.); claudio.agostinelli@unibo.it (C.A.); elena.sabattini@aosp.bo.it (E.S.); 2 Department of Medical and Surgical Sciences, University of Bologna, 40138 Bologna, Italy; maura.rossi3@unibo.it; 3 Istituto di Ematologia “Seràgnoli”, IRCCS Azienda Ospedaliero-Universitaria di Bologna, 40138 Bologna, Italy; matteo.carella3@unibo.it (M.C.); giulia.gabrielli11@unibo.it (G.G.); pierluigi.zinzani@unibo.it (P.L.Z.); 4 Dipartimento di Scienze Mediche e Chirurgiche, Università di Bologna, 40138 Bologna, Italy; 5 Pediatric Unit, IRCCS Azienda Ospedaliero-Universitaria di Bologna, 40138 Bologna, Italy; elena.facchini@aosp.bo.it (E.F.); arcangelo.prete@aosp.bo.it (A.P.); 6 Unit of Semeiotics, Liver and Alcohol-Related Diseases, IRCCS Azienda Ospedaliero-Universitaria di Bologna, 40138 Bologna, Italy; maurizio.baldassarre@unibo.it; 7 Department of Advanced Biomedical Sciences, Pathology Section, School of Medicine, University of Naples, 80131 Naples, Italy; massimo.mascolo@unina.it

**Keywords:** Hodgkin’s, adolescent, microenvironment, immune-check points

## Abstract

Background. Classical Hodgkin’s lymphoma (cHL) in adolescents between 15 and 18 years old shows a higher disease-related mortality, and the overall prognosis is worse than in both children and adults. Objectives. We investigated the immune checkpoint inhibitors (ICPIs) therapeutic targets and specific T-regulatory and cytotoxic T-cell subsets in the subgroup of adolescent cHL patients, and we challenged their prognostic power. Methods. We retrieved formalin-fixed paraffin-embedded (FFPE) tissue of adolescent patients diagnosed with cHL and tested by immunohistochemistry the immune checkpoint molecules CTLA-4, LAG-3, PD-1, and PDL1 as well as the biological markers FOXP3 and CD8. Results. All the cases of our cohort expressed the immune checkpoint molecules CTLA-4, LAG-3, and PD-1 in microenvironment (ME), and the number of PD1+ cells was strongly associated with advanced disease, being higher in stage III/IV, indicating a possible role in the progression of cHL. A higher risk of recurrence and progression occurred in patients with lower amount of CD8+ microenvironmental T-cells at diagnosis (67.14 ± 27.23 vs. 42.86 ± 17.33 *p* = 0.032 and 65.59 ± 26.68 vs. 37 ± 17.45 *p =* 0.046, respectively). Conclusions. We showed that microenvironment of cHL in adolescent patients is enriched with potential therapeutic targets of ICPI that may be considered for therapeutic applications. Furthermore, the presence of PD-1 expressing T-cells strongly relates to advanced stage disease and a low density of CD8+ T lymphocytes is associated with recurrence and progression of disease.

## Simple Summary

The 5-year survival rate of Classical Hodgkin’s lymphoma (cHL) exceeds 90%; however, adolescents between 15 and 18 years old show a higher disease-related mortality, and their overall prognosis is worse than that of both children and adults. We investigated the immune checkpoint inhibitors (ICPIs) therapeutic targets and specific T-regulatory and cytotoxic T-cell subsets in the subgroup of adolescent cHL patients, and we challenged their prognostic power. We showed that microenvironment of cHL in adolescent patients is enriched of ICPI targets that may be considered for therapeutic applications. Remarkably, LAG-3 is more consistently expressed than in pediatric and adult cHL, strengthening the state of immune anergy and reinforcing the rationale behind therapeutic approaches based on the use of new anti-LAG-3 antibodies. Furthermore, the presence of PD-1+ T-cells strongly relates to advanced stage disease, and a low density of CD8+ T lymphocytes is associated with recurrence and progression of disease.

## 1. Introduction

At present, the Classical Hodgkin’s lymphoma (cHL) 5-year overall survival rate exceeds 90% in Western countries; however, although standard chemotherapy induces a stable remission in most cases of cHL, adolescents between 15 and 18 years show a higher disease-related mortality, and their overall prognosis is worse than that of both children and adults [1,2,3,4]. A specific reason has not yet been identified with certainty: diagnostic delay, reduced participation in clinical trials, poor adherence to the treatment program, and specific social context are conditions listed as possible causes, but a certain explanation for this difference has not been clarified yet [5,6,7]. The cHL microenvironment consists of non-neoplastic inflammatory cells attracted by the large number of cytokines produced by Hodgkin and Reed-Sternberg cells (HRSCs), which favor the expansion of different immune populations and modify their functional status in order to receive pro-survival stimuli and turn off the anti-tumor immune response. To this purpose, HRSCs also shape a biological niche by organizing the spatial distribution of cells in the ME whose composition was demonstrated to influence the disease prognosis. Therefore, an accurate evaluation of the cell subsets and features of the tumor ME could represent a pivotal issue to explain the worse clinical outcomes in adolescents and might be a source of therapeutic targets. A deeper understanding of the mechanisms of tumor immune evasion allowed for the development of novel immunotherapies that act on ME cells, deplete regulatory T-cells, or re-activate T-cells. In refractory/relapsed cHL, immunotherapy with ICPI nivolumab and pembrolizumab, unlocking the PD-1 signaling pathway, has reached high response rates and stable control of the disease [8,9]. The field of immunotherapy in cHL has recently incorporated the use of newer PD-1 inhibitors, as well as anti-CTLA-4 monoclonal antibodies and anti-LAG3 (respectively ipilimumab and favezelimab), in combination with pembrolizumab or nivolumab [10,11,12,13,14,15,16,17,18]. In the present study, we investigated the ICPI therapeutic targets CTLA-4, LAG-3, PD-1, and PDL1 and the biological markers FOXP3 and CD8, which are respectively representative of specific T-regulatory and cytotoxic T-cell subsets in the subgroup of adolescent cHL patients, and we challenged their prognostic power.

## 2. Materials and Methods

### 2.1. Patients

From the archives of the Unit of Hematopathology of IRCCS S. Orsola–Bologna, we retrieved formalin-fixed paraffin embedded (FFPE) tissue samples of 36 adolescent patients diagnosed as cHL with available clinical data. All patients were treated between 2005 and 2020 in the pediatric haemato-oncology and hematology division in IRCCS Azienda Ospedaliero-Universitaria di Bologna. The study was approved by the Ethic Committees of Bologna Hospital (Code Protocol LH2004ME, number 332/2023, 0024019/23, approval date 17 May 2023).

### 2.2. Immunohistochemical Analysis

Immunostaining was carried out on whole sections of excised lymph node using BenchMark Ultra Ventana instrument and software platforms (Roche Diagnostics, Indianapolis, Indiana). Paraffin-embedded sections were dewaxed and put in antigen retrieval ULTRA CC1 (Cell Conditioner 1) (Ventana code 05424569001) at 95 °C for 16–40 min. The sections were then incubated at 36 °C with the following primary antibodies (for 16–40 min): CD8 (Ventana, Roche Diagnostics, Basel, Switzerland, rabbit monoclonal, clone SP57, code 790-4460, undiluted), FOXP3 (Abcam, Cambridge, UK, rabbit monoclonal, clone SP97, code AB99963-100, dilution 1:100), CTLA-4, (Santa Cruz, Dallas, CA, USA, mouse monoclonal, clone F-8, code sc-376016, dilution 1:80), LAG-3 (Abcam, rabbit monoclonal, clone EPR43922, code ab180187, dilution 1:8000), PD-1 (Cell Marque-Ventana, Roche Diagnostic, mouse monoclonal, clone NAT 105, code 7099029001, undilute), and PDL1 (Abcam, rabbit monoclonal, clone 28-8, code ab205921-50, dilution 1:200). The reaction was detected by the OptiView DAB Detection kit (Ventana, code 760-700). Finally, all slides were counterstained with Hematoxylin [19]. Stains were reviewed by expert hematopathologists (E.S, C.B). For each molecule observed, the average number of positive cells was calculated, evaluating 10 different high-power fields (40× HPF). PD-L1 was scored in neoplastic cells as follows: score 0 < 1% of positive cells, score 1 ≥ 1–50% of positive cells, ≥50% score 2. Quantifications of the following markers/ratio were performed: CTLA-4/FOXP3, FOXP3/CD8, and CD8/PD1.

### 2.3. Statistical Analysis

The normality of the distribution and the homogeneity of variances for continuous variables were assessed using the Shapiro–Wilk and Levene tests. Continuous variables were then reported as mean and standard deviation or median and interquartile range according to their distribution. Categorical variables were expressed as absolute frequencies and percentages. Comparisons between groups were made using Student’s *t*-test for normally distributed variables, while the Mann–Whitney U-test was used in case of significant deviations from normality. The distribution of categorical variables was compared using the chi-squared test or Fisher’s exact test. The correlation analysis was performed according to the Pearson or Spearman method as appropriate. The Receiver Operating Characteristics (ROC) curve analysis was used to determine values associated with the highest sensitivity and specificity for the outcome of interest. Survival curves according to the identified cut-offs were plotted according to the Kaplan–Meier method and compared by the Log-Rank test. All tests were two-sided, and *p*-values less than 0.05 were considered statistically significant. Analyses were performed using Stata version 18 software (StataCorp LLC, College Station, TX, USA).

## 3. Results

### 3.1. Features of the Study Population

The clinical features of the population and the therapy regimen are described in Table 1. The selected patients were adolescent, with ages ranging from 12 to 18 years (average and median age: 15 years); 55.6% were females and 44.4% males. The diagnosis was formulated on excised lymph nodes in 36/36 cases (100%). The cHL histological subtypes distribution was: 33/36 (91.7%) nodular sclerosis (SN), 2/36 (5.5%) mixed cellularity (CM), 1/36 (2.8%) lymphocyte depleted (LD). Twenty-four cases were diagnosed at stage II of disease (66.7%), while 9/36 (25%) and 3/36 (8.3%) were stage III and stage IV, respectively. Nine patients (25%) had B-symptoms (fever and/or weight loss and/or sweating) and extra-nodal involvement was present in 4/36 (11.8%) of the cases (2 lung, 1 pericardium, and 1 osteolytic bone lesions). Patients were treated as follows: LH2004 protocol including 6/4COPP/ABV or 3/ABVD in 17/36 (47.2%) patients, EuroNet PHLC2 protocol 2 including OEPA+1/4COPDAC in 6/36 (16.7%) patients, or ABVD scheme in 13/36 (36.1%) patients. Seven patients experienced recurrence (19.4%) of disease, 4 progressed (11.1%), and 3 (8.3%) patients died of disease (see complete data in Table 1).

### 3.2. Biologic Profiling

**Figure 1:**
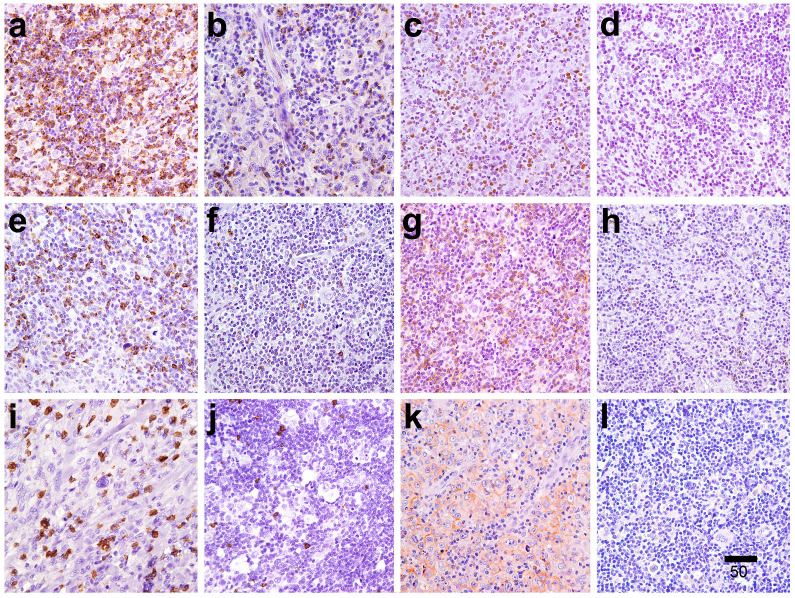
In the pictures were reported cases with (**a**) high and (**b**) low expression of the immune histochemical markers CTLA-4, (**c**) high and (**d**) low expression of FOXP3, (**e**) high and (**f**) low expression of LAG-3, and (**g**) high and (**h**) low expression of PD-1 in the early and advanced stages of disease, respectively, and (**i**) high and (**j**) low expression of CD8 in a patient who experienced a recurrence and PDL1 expression (**k**) score 2 and (**l**) score 0 (400×). (Scale bar 50 μm).

Complete immunohistochemical markers profiling was reported in Table 2. All the 36 cases of our cohort expressed the immune checkpoint molecules CTLA-4, LAG-3, and PD-1 in ME cells. CTLA-4 was the most represented (456.06 ± 280.86), followed by LAG-3 [68.00 (51–94)] and PD-1 [37 (24–58)] (Table 2; Figure 1). The positivity was granular and cytoplasmatic for CTLA-4 and LAG-3, and membrane for PD-1. The T-reg differentiation molecule FOXP-3 showed nuclear staining in many ME elements in all samples (Figure 1). We found a strong linear correlation (*p* < 0.001) between the expression of CTLA-4 and FOXP3 (see Supplementary Material). A variable subset of CD8^+^ lymphocytes was observed in each case (62 ± 27.20). PD-L1 was scored in neoplastic cells as follows: score 0 in 7 (20%), score 1 in 15 (42.95), and score 2 in 13 (37.1%). A higher FOXP3/CD8 ratio was associated with progression [1.76 (0.88–2.67) vs. 2.92 (2.76–5.35)] (*p* = 0.030), whereas a higher CD8/PD-1 ratio was related to a lower stage of disease [stage II 1.53 (1.11–4.00) vs. stage III/IV 0.92 (0.75–1.82)] (*p* = 0.03) (Complete analysis was reported in Supplementary Material). The 2 cases classified as mixed cellularity showed expression of LAG-3, CTLA-4, PD-1, and CD8 above the mean and median values and low score positivity for PD-L1 (score 0 and 1), whereas FOXP-3 showed discordant values in the two cases, resulting in one being above average and another below average. The lymphocyte depletion case, on the other hand, was characterized by widespread PD-L1 expression, numbers of LAG-3+, CTLA-4+, FOXP3+, and CD8+ cells below mean/median values and a fair proportion of PD1+ elements above mean/median values.

### 3.3. Clinical/Biologic Correlations

**Figure 2:**
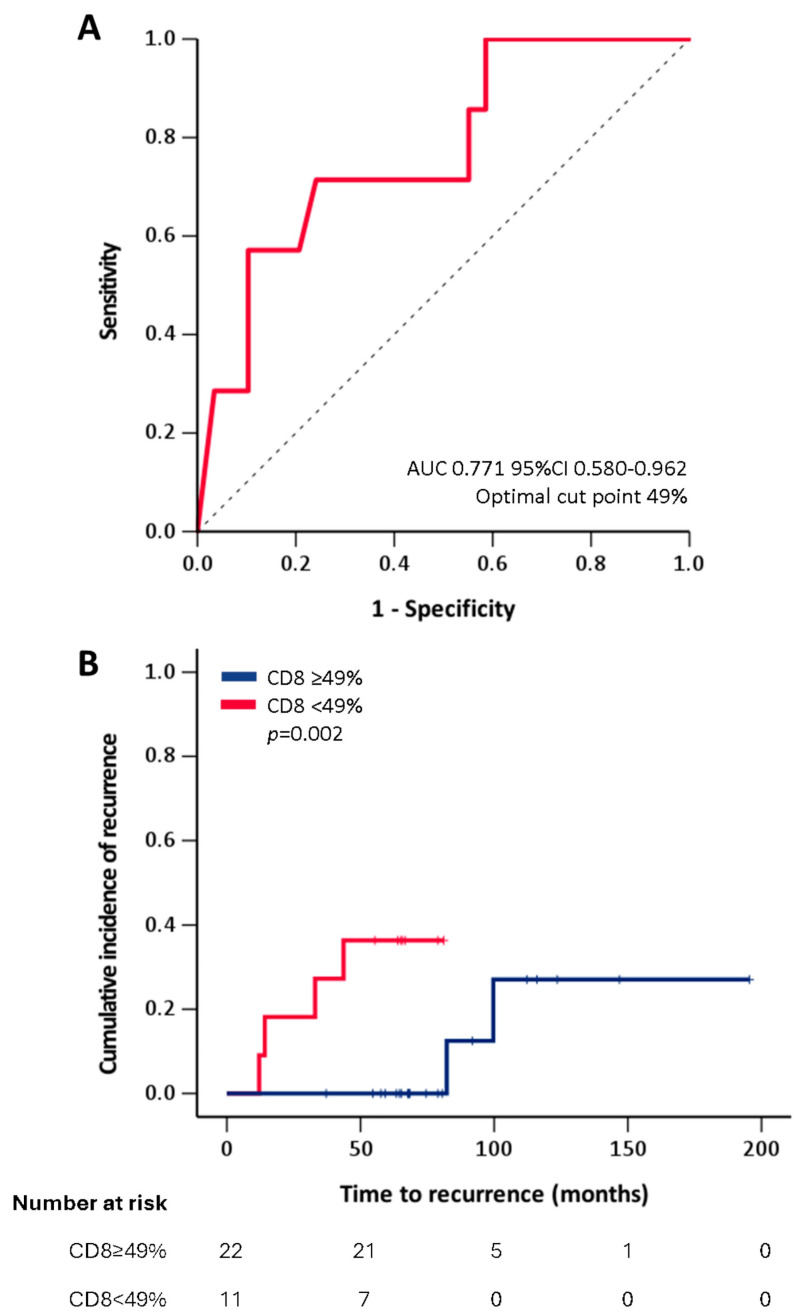
Receiver Operating Characteristics (ROC) curve analysis showing sensitivity and 1-specifity of percentage of CD8 positive cells versus disease recurrence (Panel (**A**)). Cumulative incidence of recurrence in patients with CD8 positive cells lower or higher than the cut-off (49%) identified in the ROC curve analysis (Panel (**B**)).

When we investigated the correlations between biologic parameters, clinical data, and outcome extra-nodal disease, stage and high LDH level statistically correlated to a higher risk of progression of disease (*p* = 0.009, *p* = 0.005, and 0.048, respectively). As expected, patients that recurred had a higher risk of progression and death from disease (*p =* 0.003 and <0.001, respectively, Supplementary Table S1). The amount of PD1+ cells was significantly associated with the stage of the disease (*p* < 0.001) being higher in stage III/IV [64 (53–107)] versus stage II [29.50 (20–47)] (*p* = 0.001). Furthermore, a higher risk of recurrence and progression occurred in patients with lower amount of CD8+ microenvironmental T-cells at diagnosis (67.14 ± 27.23 vs. 42.86 ± 17.33 *p* = 0.032 and 65.59 ± 26.68 vs. 37 ± 17.45 *p* = 0.046, respectively) (Figure 2). The complete statistical analysis is available in Supplementary Tables S1–S3. The association between the number of CD8 positive cells and disease recurrence was further investigated by Kaplan–Meier analysis, in which patients were classified according to the cut-off of 49 positive (40× HPF), as identified by the ROC curve analysis (Figure 2A). As shown in Figure 2B, the cumulative incidence of recurrence was significantly lower in subjects with CD8+ cells ≥ 49 (40× HPF) (*p* = 0.002). The comparison with the data concerning the same markers in children and adult series in previous studies is shown in Table 3.

## 4. Discussion

In the present study, we investigated the ICPI therapeutic targets CTLA-4, LAG-3, PD-1, and PDL1 and the biological markers FOXP3 and CD8, representative of specific T-regs and cytotoxic T-cells in a cohort of 36 adolescent cHL patients, challenging their prognostic power. Recent discoveries on the dynamics of the Hodgkin’s microenvironment have led to the development of new immunotherapies for drug-resistant patients that experience progressive disease or relapse [30]. The ICPI nivolumab and pembrolizumab has reached high response rates and stable control of disease [8,9]; however, newer PD-1 inhibitors as well as anti-CTLA-4 and anti-LAG3 antibodies, in combination with nivolumab or pembrolizumab, have been recently incorporated in the field of immunotherapy [8,9,10,11,12,13,14,15,16,17,18].

All the cases of our cohort expressed the immune checkpoint molecules CTLA-4, PD-1, and LAG-3 in ME cells. Interestingly, CTLA-4 was more represented than LAG-3 and PD-1, which was in line with studies which observed a high number of CD4+/CTLA-4+ T-cells in the ME in 22 adult patients [31]. We could not confirm any correlation between CTLA-4+ cell number and prognosis, as recently reported in 40 adult cHL cases by Pangaribuan et al., who showed its association with IPS adverse prognostic factors in cases of advanced-stage cHL, suggesting that immune checkpoints might be involved in cancer progression [27]. Despite CTLA-4+ lymphoid subsets being identified as key players in cHL ME in several studies, few clinical trials have evaluated the effects of the combination of anti-CTLA-4 antibody (ipilimumab) with nivolumab or nivolumab and BV [15]. A phase II study is currently ongoing assessing BV and nivolumab with or without ipilimumab in patients with R/R HL (NCT01896999).

CTLA-4 selectively identifies FOXP3+ regulatory cells, and we confirmed a strong linear correlation between the expression of CTLA-4 and FOXP3; nonetheless, the number of FOXP3+ cells was lower than those expressing CTLA4. Indeed, Aoki et al. demonstrated that, in cHL ME CTLA-4+/FOXP3-, T-cells outnumber the CTLA-4+/FOXP3+ ones, and that they are increased when in proximity or in contact to HRSCs and to tumor-associated macrophages (TAM). The CTLA-4+/FOXP3- cells are frequently PD-1- and engage CD86, the cognate ligand for CTLA-4, expressed by HRSC and TAM [31].

High levels of FOXP3 positive cells were significantly associated with better PFS and longer OS, as reported by Alvaro et al., Chateille et al., and our group [25,28,29] in adult cHL patients, but this observation was not confirmed in our series of patients.

LAG-3 acts synergistically with PD-1 and/or CTLA-4 to limit T-cell expansion, and LAG-3+ T-cells inhabit cHL ME [31,32,33,34]. A distinct CD4+/LAG-3+/FOXP3− T-cell subset, expressing IL-10 and TGFβ, was recently identified in cHL and could represent a Th1-type T-reg population, able to suppress effector CD8+ T-cell function in cHL [31]. LAG-3 expression was observed in cHL MEs of a subset of pediatric patients, with conflicting results regarding its prognostic role: Jimenez et al. reported an inferior EFS when both PD-1 and LAG-3 molecules were present [24], while in the study of Moerdler et al., patients with the lowest expression density had the worst EFS, and those with the highest showed the best EFS [26]. Furthermore, LAG-3 was reported to be expressed in the MEs of a subgroup (65%) of adult cHL patients [35]. For the first time, we herein demonstrated that LAG-3+ cells were always present in cHL MEs in adolescents, though without a statistically significant association with prognosis. This may represent the biologic rationale for targeting LAG-3 in combination with anti-PD-1 antibodies in the treatment of relapsed/refractory cHL. Indeed, the LAG-3 and PD-1 co-blockade molecule favezelimab—a humanized immunoglobulin (Ig) G4 LAG-3 inhibitor—is being explored in combination with pembrolizumab (NCT03598608) in a multi-cohort phase I/II study for patients with R/R hematologic tumors [16,18]. A clinical study investigating the preliminary efficacy of the anti-LAG-3 monoclonal antibody BMS-986016 in combination with nivolumab in relapsed or refractory cHL is currently ongoing (clinicaltrials.gov identifier: 02061761). Furthermore, Tebotelimab, a bispecific PD-1×LAG-3 DART molecule that inhibits both PD-1 and LAG-3, was investigated for clinical safety and activity in a phase 1 dose-escalation and cohort-expansion clinical trial in adult patients with solid tumors or hematologic malignancies with disease progression on previous treatment (ClinicalTrials.gov identifier: NCT03219268) [36].

In our adolescent series, the number of PD1+ cells was strongly associated with advanced disease, being higher in stage III/IV, indicating a possible role in the progression of cHL, although, unlike other authors in adults, we did not identify significant statistical association with a worse prognosis [25]; however, PD-1 was expressed in cHL ME, confirming itself as a therapeutic target also in adolescent settings. We found a protective role of a high CD8+ lymphocyte count in cHL ME: patients that recurred or experienced progression had lower amounts of CD8+ cells at diagnosis, as well as a high FOXP3/CD8 ratio, while a CD8+-enriched ME characterizes low stage of disease as compared to a ME enriched with exhausted PD1+ T-cells.

Only in a few previous studies has the number of intra-tumoral CD8+ T-cells in cHL patients been associated with outcome. Alonso-Álvarez et al. showed that high CD4+ and low CD8+ T-cells infiltrates in tumor specimens were associate with poor prognosis in cHL adult patients [23], and, recently, Michot et al. found immune CD8+ depletion and overexpression of LAG-3 on CD4+ helper T-cells as possible mechanisms of resistance to immunotherapies in patients with cHL [37]. The favorable prognostic significance of the high number of CD8+ cells in our court should not be surprising given the key role of cytotoxic lymphocytes in antitumor immunity, although this may not apply to all cases. In fact, since CD8+ T-cells interact with HRS cells via MHC class I, the presence of β2-microglobulin (β2M) inactivating mutations, which disrupt the expression of the β2M/MHC class I dual protein complex at the HRSC surface, may hide HRSC to activated CD8+ cytotoxic lymphocytes [38]. As a matter of fact, only in 20% of cHL cases does HRSC show positive immunohistochemical membrane staining for β2M or MHC class I proteins. Furthermore, atypical functional phenotypes of CD8+ cell were reported in cHL: Le KS et al. demonstrated a CD8 T-cell differentiation pathway leading to the acquisition of some T-Follicular Helper similarities [39].

## 5. Conclusions

In summary, our study is the first to have investigated the cHL microenvironment of adolescents, which appears to have characteristics and dynamics partly similar and partly different compared to those of children and adults. We showed that microenvironment of cHL in adolescent patients, as in children and adults, is enriched with potential therapeutic targets of ICPI that may be considered for therapeutic applications, with CTLA-4 being the most represented. However, remarkably, LAG-3 is more consistently expressed, reinforcing the rationale for therapeutic approaches based on the use of new anti-LAG-3 antibodies. Although not associated with recurrence in both children and adult series, the presence of PD-1 expressing T-cells strongly relates to advanced-stage disease. Similarly to adults, low density of CD8+ T lymphocytes in adolescents is associated with worse prognosis, but high numbers of FOXP3+ T-regs do not positively impact disease progression.

## Figures and Tables

**Table 1:** Clinical features of the population and therapy regimen. Data are reported as mean ± standard deviation, median and IQR or absolute number and percentage (%).

Study Cohort: 36 Patients	
Age (years)	15.22 ± 1.71
Sex (female)	20 (55.6%)
Stage II/III/IV	24 (66.7%)/9 (25%)/3 (8.3%)
Extra-nodal involvement	4 (11.1%)
First line treatment response	29 (80.6%)
Second line treatment response	4 (57.1%)
Recurrence	7 (19.4%)
Progression	4 (11.1%)
Death	3 (8.3%)
LDH (U/l)	215.50 (160–269)

**Table 2:** Immunohistochemical results. Data are reported as mean ± standard deviation, median and IQR or absolute number and percentage (%).

Marker	Distribution
PDL1	score 0: 7 (20%) score 1: 15 (42.9%) score 2: 13 (37.1%)
CD8	62.42 ± 27.20
PD-1	37 (24–58)
LAG-3	68 (51–93)
CTLA-4	456.06 ± 280.86
FOXP3	126.44 ± 86.25
CTLA-4/FOXP3	3.69 (2.36–6.08)
FOXP3/CD8	1.90 (1.01–2.88)
CD8/PD-1	1.40 (0.91–2.53)

**Table 3:** Data concerning clinical impact of biologic markers in children and adult series in previous studies.

Marker	Children (<12 Years)		Adults (>18 Years)	
	Significance	Reference	Significance	Reference
PDL1	no impact on prognosis	Dilly-Feldis et al. [20]	high PD-L1 related to shorter PFS	Romer et al. [21]
CD8	no impact on prognosis	Barros et al. [22]	high CD8 related to longer FFTF	Alonso et al. [23]
PD-1	high PD-1 related to inferior EFS	Jimenez et al. [24]	high PD1 related to shorter PFS	Agostinelli et al. [25]
LAG-3	high LAG-3 related to inferior EFS	Jimenez et al. [24]	no data on this specific sub group	
	high LAG-3 related to longer EFS	Moerdler et al. [26]		
CTLA-4	no data on this specific sub group		high CTLA-4 associated with IPS	Pangaribuan et al. [27]
FOXP3	no data on this specific sub group		high FOXP3 related to longer PFS/OS	Alvaro et al. [28]
			high FOXP3 related to longer PFS/OS	Agostinelli et al. [25]
			high FOXP3 related to longer OS	Chetaille et al. [29]
CTLA-4/FOXP3	no data on this specific sub group		no data on this specific sub group	
FOXP3/CD8	lower ratio related to improved outcome	Barros et al. [22]	no data on this specific sub group	
CD8/PD-1	no data on this specific sub group		no data on this specific sub group	

PFS: progression free survival; FFTF: freedom from treatment failure; OS: overall survival; IPS: international prognostic score.

## Data Availability

The original contributions presented in this study are included in the article/Supplementary Material. Further inquiries can be directed to the corresponding author.

## Supplementary Materials

The following supporting information can be downloaded at: https://www.mdpi.com/article/10.3390/cancers16244210/s1, Table S1. Anthropometric, clinical and biological profile of patients included in the study according to the presence of disease recurrence; Table S2. Anthropometric, clinical and biological profile of patients included in the study according to the presence of disease progression; Table S3. Anthropometric, clinical and biological profile of patients included in the study according to the stage of the disease.
