# Correction: Bertuzzi et al. Microenvironmental Traits of Classical Hodgkin’s Lymphoma in Adolescents and Their Prognostic Impact. *Cancers* 2024, *16*, 4210

**DOI:** 10.3390/cancers17030429

**Published:** 2025-01-27

**Authors:** Clara Bertuzzi, Simona Righi, Giovanna Motta, Maura Rossi, Matteo Carella, Giulia Gabrielli, Elena Facchini, Maurizio Baldassarre, Arcangelo Prete, Pier Luigi Zinzani, Massimo Mascolo, Claudio Agostinelli, Elena Sabattini

**Affiliations:** 1 Haematopathology Unit, IRCCS Azienda Ospedaliero-Universitaria di Bologna, 40138 Bologna, Italy; clara.bertuzzi@aosp.bo.it (C.B.); giovanna.motta@aosp.bo.it (G.M.); claudio.agostinelli@unibo.it (C.A.);elena.sabattini@aosp.bo.it (E.S.); 2 Department of Medical and Surgical Sciences, University of Bologna, 40138 Bologna, Italy; maura.rossi3@unibo.it; 3 Istituto di Ematologia “Seràgnoli”, IRCCS Azienda Ospedaliero-Universitaria di Bologna, 40138 Bologna, Italy; matteo.carella3@unibo.it (M.C.); giulia.gabrielli11@unibo.it (G.G.); pierluigi.zinzani@unibo.it (P.L.Z.); 4 Dipartimento di Scienze Mediche e Chirurgiche, Università di Bologna, 40138 Bologna, Italy; 5 Pediatric Unit, IRCCS Azienda Ospedaliero-Universitaria di Bologna, 40138 Bologna, Italy; elena.facchini@aosp.bo.it (E.F.); arcangelo.prete@aosp.bo.it (A.P.); 6 Unit of Semeiotics, Liver and Alcohol-Related Diseases, IRCCS Azienda Ospedaliero-Universitaria di Bologna, 40138 Bologna, Italy; maurizio.baldassarre@unibo.it; 7 Department of Advanced Biomedical Sciences, Pathology Section, School of Medicine, University of Naples, 80131 Naples, Italy; massimo.mascolo@unina.it

Massimo Mascolo was not included as an author in the original publication [1]. The corrected Author Contributions Statement appears here. The authors apologize for any inconvenience caused and state that the scientific conclusions are unaffected. This correction was approved by the Academic Editor. The original publication has also been updated.

**Author Contributions:** Conceptualization, E.S. and C.B.; methodology, E.S. and S.R.; software, M.B.; validation, G.M., S.R. and M.R.; formal analysis, S.R.; investigation, S.R., E.S., C.B., E.F., M.C., G.G. and M.B.; data curation, S.R.; writing—original draft preparation, C.A.; writing—review and editing, S.R. and M.M.; supervision, E.S., A.P. and P.L.Z.; funding acquisition, E.S. All authors have read and agreed to the published version of the manuscript.
