# Supplementary material for: Microenvironmental Traits of Classical Hodgkin’s Lymphoma in Adolescents and Their Prognostic Impact
Source: Cancers (Basel). 2024 Dec 18;16(24):4210. doi: 10.3390/cancers16244210 (PMC11674618; doi:10.3390/cancers16244210)
Supplement: Supplementary file 1 [file cancers-16-04210-s001.zip › cancers-3341000-supplementary.pdf]

Supplementary Table S1. Anthropometric, clinical and biological profile of patients included in the study according to the presence of disease recurrence. Data is reported as mean and standard deviation, median and IQR or absolute number and percentage.

	No recurrence N=29	Recurrence N=7	p-value
age	15.28 ± 1.79	15.00 ± 1.41	0.707
Sex (female)	17 (58.6%)	3 (42.9%)	0.451
Stage			
II	18 (62.1%)	6 (85.7%)	0.221
III	9 (31.0%)	0 (0.0%)	
IV	2 (6.9%)	1 (14.3%)	
Extra-nodal involvement	3 (10.3%)	1 (14.3%)	0.766
First line treatment response	25 (86.2%)	4 (57.1%)	0.081
Second line treatment response	3 (60.0%)	1 (50.0%)	0.809
Progression	1 (3.4%)	3 (42.9%)	0.003
Death	0 (0.0%)	3 (42.9%)	<0.001
PDL1 expression			
score 0	5 (17.9%)	2 (28.6%)	0.665
score 1	13 (46.4%)	2 (28.6%)	
score 2	10 (35.7%)	3 (42.9%)	
LDH (U/L)	216 (169-240)	205 (146-289)	0.789
CD8	67.14 ± 27.23	42.86 ± 17.33	0.032
PD-1	45 (24-64)	29 (24-58)	0.603
LAG-3	65 (52-112)	71 (49-86)	0.603
CTLA-4	472.04 ± 298.90	437.14 ± 209.33	0.774
FOXP3	117.72 ± 71.58	162.57 ± 132.63	0.222
CTLA-4/FOXP3	3.83 (2.49-6.11)	3.42 (2.10-5.17)	0.592
FOXP3/CD8	1.79 (1.10-2.59)	3.00 (0.91-7.12)	0.069
CD8/PD-1	1.40 (0.96-2.95)	1.17 (0.83-2.39)	0.378

Supplementary Table S2. Anthropometric, clinical and biological profile of patients included in the study according to the presence of disease progression. Data is reported as mean and standard deviation, median and IQR or absolute number and percentage.

	No progression N=32	Progression N=4	p-value
age	15.22 ± 1.74	15.25 ± 1.71	0.973
Sex (female)	18 (56.2%)	2 (50.0%)	0.813
Stage			
II	22 (68.8%)	2 (50.0%)	0.005
III	9 (28.1%)	0 (0.0%)	
IV	1 (3.1%)	2 (50.0%)	
Extra-nodal involvement	2 (6.2%)	2 (50.0%)	0.009
First line treatment response	28 (87.5%)	1 (25.0%)	0.003
Second line treatment response	3 (60.0%)	1 (50.0%)	0.809
Recurrence	4 (12.5%)	3 (75.0%)	0.003
Death	0 (0.0%)	3 (75.0%)	<0.001
PDL1 expression			
score 0	5 (16.1%)	2 (50.0%)	0.152
score 1	13 (41.9%)	2 (50.0%)	
score 2	13 (41.9%)	0 (0.0%)	
LDH (U/L)	205 (159-238)	289 (269-431)	0.048
CD8	65.59 ± 26.68	37.00 ± 17.45	0.046
PD-1	45 (23-58)	31 (27-74)	0.957
LAG-3	65 (51-101)	75 (59-86)	0.960
CTLA-4	475.94 ± 293.59	380.75 ± 146.05	0.532
FOXP3	123.84 ± 86.17	147.25 ± 96.99	0.616
CTLA-4/FOXP3	3.98 (2.37-6.13)	2.76 (1.99-4.30)	0.351
FOXP3/CD8	1.76 (0.88-2.67)	2.92 (2.76-5.35)	0.030
CD8/PD-1	1.40 (0.94-3.38)	1.17 (0.53-1.72)	0.229

Supplementary Table S3. Anthropometric, clinical and biological profile of patients included in the study according to the stage of the disease. Data is reported as mean and standard deviation, median and IQR or absolute number and percentage.

	Stage II N=24	Stage III/IV N=12	p-value
age	15.12 ± 1.78	15.42 ± 1.62	0.636
Sex (female)	14 (58.3%)	6 (50.0%)	0.635
Extra-nodal involvement	1 (4.2%)	3 (25.0%)	0.061
First line treatment response	20 (83.3%)	9 (75.0%)	0.551
Second line treatment response	1 (33.3%)	3 (75.0%)	0.270
Recurrence	6 (25.0%)	1 (8.3%)	0.234
Progression	2 (8.3%)	2 (16.7%)	0.453
Death	2 (8.3%)	1 (8.3%)	1.000
PDL1 expression			
score 0	4 (17.4%)	3 (25.0%)	0.301
score 1	12 (52.2%)	3 (25.0%)	
score 2	7 (30.4%)	6 (50.0%)	
LDH (U/L)	222 (173-289)	174 (159-226)	0.136
CD8	59.50 ± 28.95	68.25 ± 23.37	0.370
PD-1	30 (20-47)	64 (53-107)	0.001
LAG-3	68 (50-93)	68 (56-102)	0.557
CTLA-4	482.70 ± 244.00	431.25 ± 350.49	0.614
FOXP3	139.67 ± 94.50	100.00 ± 62.06	0.198
CTLA-4/FOXP3	3.50 (2.37-6.08)	4.94 (1.68-5.95)	0.627
FOXP3/CD8	2.08(1.43-3.18)	1.55 (0.67-2.50)	0.149
CD8/PD-1	1.53 (1.11-4.00)	0.92 (0.75-1.82)	0.033
